# An exploratory cluster randomised trial of a university halls of residence based social norms marketing campaign to reduce alcohol consumption among 1st year students

**DOI:** 10.1186/1747-597X-8-15

**Published:** 2013-04-18

**Authors:** Graham F Moore, Annie Williams, Laurence Moore, Simon Murphy

**Affiliations:** 1 DECIPHer, School of Social Sciences, Cardiff University, 1-3 Museum Place, Cardiff CF10 3BD, UK

## Abstract

**Aims:**

This exploratory trial examines the feasibility of implementing a social norms marketing campaign to reduce student drinking in universities in Wales, and evaluating it using cluster randomised trial methodology.

**Methods:**

Fifty residence halls in 4 universities in Wales were randomly assigned to intervention or control arms. Web and paper surveys were distributed to students within these halls (n = 3800), assessing exposure/contamination, recall of and evaluative responses to intervention messages, perceived drinking norms and personal drinking behaviour. Measures included the Drinking Norms Rating Form, the Daily Drinking Questionnaire and AUDIT-C.

**Results:**

A response rate of 15% (n = 554) was achieved, varying substantially between sites. Intervention posters were seen by 80% and 43% of students in intervention and control halls respectively, with most remaining materials seen by a minority in both groups. Intervention messages were rated as credible and relevant by little more than half of students, though fewer felt they would influence their behaviour, with lighter drinkers more likely to perceive messages as credible. No differences in perceived norms were observed between intervention and control groups. Students reporting having seen intervention materials reported lower descriptive and injunctive norms than those who did not.

**Conclusions:**

Attention is needed to enhancing exposure, credibility and perceived relevance of intervention messages, particularly among heavier drinkers, before definitive evaluation can be recommended. A definitive evaluation would need to consider how it would achieve sufficient response rates, whilst hall-level cluster randomisation appears subject to a significant degree of contamination.

**Trial registration:**

ISRCTN: ISRCTN48556384

## Introduction

Excessive alcohol consumption among university students has been linked to educational difficulties, psychosocial problems, antisocial behaviours, injuries and risky sexual behaviours [1]. However, heavy alcohol consumption is perceived as a rite-of-passage by students in the United Kingdom (UK) [2], playing a central role in the construction of student identities [3,4], with university students drinking more than peers who enter the workforce [5]. One survey found that half of 1st year students exceeded recommended weekly drinking limits [6], while a third did so into their 3rd year. Recent policy efforts to increase proportions of young people entering university have perhaps exposed more young people than ever to an environment where hazardous consumption of alcohol is widespread. Interventions in university settings are therefore increasingly seen as important in reaching young people at risk from hazardous drinking. However, much of the limited evidence to inform policy and practice comes from the US, where purchasing alcohol is an illicit behaviour for most undergraduates, with consumption lower than among UK students [5,7].

One approach to reducing student drinking which shows promise in recent (largely US-based) studies is the social norms approach [8]. Social norms are central to a range of models in health psychology. For example, Social Learning Theory emphasises roles of descriptive norms (perceived behaviours of others) in shaping behaviours [9], while the Theory of Planned Behaviour emphasises roles of injunctive norms (perceived social approval of the behaviour) in forming behavioural intentions [10]. While most social norms interventions have emphasised descriptive norms (e.g. communicating that drinking isn’t as prevalent as students think), prospective research has shown injunctive and descriptive norms to be independent predictors of student drinking [11].

Advocates of social norms interventions focus upon the fallibility of normative perceptions, arguing that people overestimate the prevalence of unhealthy behaviours among peers. Overestimation of peer alcohol consumption amongst students has been widely reported [12-16], including one UK study [17]. Social norms interventions aim to correct misperceptions through mailed, web-based or face-to-face feedback on peer drinking norms [18,19], or social marketing campaigns [20,21]. A 2009 Cochrane review [8] concluded that web-based or one-to-one feedback reduced consumption, though mailed or group feedback were ineffective, with findings for social norms marketing campaigns equivocal.

Caution should however be exercised in applying these findings to the UK. Given the high levels of consumption of alcohol in many UK universities, communicating ‘actual’ drinking norms may perversely reinforce hazardous norms, whilst attempts to persuade students that heavy drinking is not the norm may lack credibility. At the time of the study, only one UK Randomised Controlled Trial (RCT) had evaluated impacts of a social norms intervention on student drinking, with students assigned to the intervention group significantly more likely to drop out of the study, perhaps indicating limited engagement with the intervention [18]. More recently, and in contrast to the conclusions of the earlier Cochrane review based largely on US evidence, one trial in 22 UK universities found no evidence of effects of web-based normative feedback on student drinking [22].

Despite limited UK evidence, plans for a social norms intervention in universities in Wales were announced in a 2010 report from the Cabinet Office Behavioural Insights Team [23]. A study was then commissioned to conduct a survey to inform the development of key messages, and a pragmatic evaluation of the intervention. First year students represent a priority group due to higher risk of hazardous drinking [6]. Whilst social norms studies have often focused on perceived norms in relation to a ‘typical student’, perceived norms of more specific groups have been shown to correlate more strongly with drinking behaviour [14]. Hence, intervention targeted first year students, with perceived norms relating to other first years, the same sex and in the same university. Halls of residence were selected as a distribution channel due to the high percentage of first years in Wales accommodated within them.

Though social norms interventions are typically evaluated with little exploration of the settings in which they are delivered, attempts to change behaviours without considering the contexts in which they are formed, are likely to be of limited and variable success [24,25]. For example, one US based intervention, Project Northland, a community intervention to reduce alcohol misuse in adolescents, achieved promising impacts in rural settings [26], though weaker impacts in urban settings, with actions of the intervention perhaps drowned out by the multitude of pro-alcohol stimuli in the urban environment [27]. Hence, in the present evaluation, the social norms campaign was accompanied by a university-wide toolkit, with universities supported by a project officer in reviewing alcohol-related policies and practices, and implementing evidence-informed changes [28]. The development and implementation of this toolkit will be described in detail elsewhere.

This paper presents findings from the exploratory trial of the social norms campaign. Medical Research Council guidelines for developing and evaluating complex interventions emphasise the importance of exploring uncertainties in the implementation of the intervention and proposed methods of evaluation [29] prior to definitive evaluation. Hence, this exploratory trial aimed to inform decisions on whether, and how, to proceed to definitive evaluation, by addressing a number of research questions.

A key challenge in evaluating university-based interventions is achieving sufficient responses to be confident of representativeness. In the only peer-reviewed survey of drinking norms in a UK university, a response rate of 4% was achieved to a web-based survey [17]. The present study adopts web and paper based survey methods in order to boost responses. Deriving suitable comparison groups is also challenging for social marketing based campaigns, with many using cluster allocation but individual-level analysis, producing potentially spurious results [30]. In deriving comparison groups, there is perhaps a trade-off between the costs associated with a higher level unit of allocation and the risk of contamination. Randomising at the university level would mean that large numbers of universities would be required hence increasing evaluation costs, while randomisation at a lower level such as halls of residence may reduce cost, though increases the risk of contamination. The feasibility or contamination associated with cluster randomisation at the residence hall level will be explored in this study.

For a campaign to be successful, the target audience must not only be exposed to messages and understand them, but must find them credible and relevant [31]. Hence, this study quantifies the percentage of students exposed to a social norms intervention, as well as recall, perceived credibility and relevance of key messages. Perceived norms are one of a multiplicity of influences on drinking, at multiple socio-ecological levels [24], and hence changes in perceived norms are perhaps unlikely to be matched by behavioural changes of the same magnitude. Whilst this study will measure behavioural outcomes, it will likely be underpowered to detect small behavioural changes. Hence, the study will primarily will examine impacts upon hypothesised mediators of change (i.e. perceived descriptive and injunctive norms).

A key weakness of previous social norms evaluations is that outcomes are invariably based on self-report [8]. As described, social norms interventions aim to change behaviour through highlighting the undesirability of excessive drinking. Experimental studies indicate that communicating the undesirability of excessive drinking through exposing students to information about peer norms leads to biased estimates of students’ own consumption [32]. Hence, these studies may have provided an over-optimistic view of behavioural impacts, with lower estimates in intervention arms arguably reflecting enhanced social desirability bias, rather than behavioural change. This study will therefore examine the acceptability of requesting hair samples as an objective method of quantifying alcohol consumption in any future definitive trial. While hair samples have shown potential as a means of measuring alcohol consumption, allowing detection of alcohol volumes consumed in the weeks prior to testing [33], the feasibility of this method in large scale studies, or with student populations, has been little explored.

## Methods

### The social norms intervention

The intervention is a social norm marketing campaign to correct misperceptions regarding behaviours and social expectations of peers among first year students, and thus influence alcohol consumption and alcohol related behaviours. Key messages (developed via survey of first year students in the same universities conducted in 2011) were delivered via placement of posters, drinks mats (coasters), drinking glasses, meal planners (i.e. 7-day calendar on which students can plan their meals for the week) and mirror stickers in halls of residence in 4 universities (see Table 1). Drinks mats and glasses were placed in self-catering kitchens within residence halls. All messages highlighted discrepancy between students’ perceptions of the behaviours (descriptive norms) or social expectations of others (injunctive norms) based on findings from an earlier survey. Messages emphasised both overestimation of risk behaviours (e.g. alcohol consumption) and underestimation of protective behaviours (e.g. including soft drinks in a night out). While most previous social norms interventions have involved communicating exact levels of drinking (e.g. ‘xx% of students drink xx times/units per week or less’), in the Welsh sample, while reported drinking was lower than perceived norms, drinking levels were hazardous. That is, an average drinking occasion lay on the border of NHS definitions of binge drinking (8 units for men/6 for women), while most students did report drinking more than recommended weekly limits (21 units for men, 14 for women). Hence, communicating absolute values for drinking would risk further normalising hazardous drinking. A decision was therefore made to emphasise discrepancy rather than present absolute values. The intervention also differed from many social norms interventions in that it attempted to prevent uptake of risky behaviours rather than changing established patterns, with intervention messages communicated to students from entry to university onwards. Fuller details can be found in the published study protocol [28].

**Table 1:** Social norms intervention components and examples of core messages communicated within them

**Timing**	**Material**	**Core message**
October 2011	Posters	'Those around you are drinking less than you think: students overestimate what others drink by 44%'
		'Most of us significantly overestimate the amount that others drink'
	Beer mats/coasters	'Those around you are drinking less than you think: students overestimate what others drink by 44%'
		'Most of us significantly overestimate the amount that others drink'
	Window stickers	'Few of us approve of people who drink to the point of losing it'
January 2012	Posters	'Most students drink to feel confident, but 70% have embarrassed themselves when drunk'
	Drinking glasses	'Time for a break? Many students limit their drinking by including soft drinks in the night'
	Gender specific leaflets	Males: '86% of Males have never damaged their halls of residence when drunk'
		Females: 'How much do you think the average female first year student drinks? Halve it. It really is less than you think.'

### Sampling and recruitment

Six universities in Wales were invited to take part, of whom 4 agreed. Participants were 1st year undergraduates in university maintained halls of residence (n = 50) within these four universities (approximately 3800 eligible students).

### Measures

#### Demographics

Students were asked to indicate their sex, age, course, ethnicity, home/international status, and which hall they lived in.

#### Intervention exposure/contamination, recall, evaluative responses and perceived impacts

For each material (posters, drinks mats, glasses), students were asked to indicate whether they had seen that material i) in their own hall of residence or ii) in another student’s hall of residence. Students were also asked to indicate whether they had been exposed to competing pro-alcohol communications in relation to i) happy hours, ii) on-campus student drinking nights (e.g. drink the bar dry) or iii) student drinking nights in off-campus bars and clubs.

To assess recall of intervention messages, those indicating having seen at least one material were presented with a list of messages, some of which were semantically consistent with messages in the campaign (e.g. other students drink less than you think) and some of which were not (e.g. other students drink more than you think), and asked to indicate whether they believed the message formed part of the campaign. Students were asked to indicate their level of agreement on a 5-point likert scale (strongly disagree to strongly agree) that materials were believable, relevant, changed their perceptions of other students’ drinking, made them more conscious of moderating the impacts of alcohol, and made them limit their own alcohol consumption.

#### Perceived drinking norms

Descriptive norms were evaluated using the Drinking Norms Rating Form (DNRF); a modified version of the Daily Drinking Questionnaire on which students are asked to estimate how much a typical member of a reference group drinks on each day of a typical week. Injunctive norms were evaluated using 3 items of a 4-item scale previously used by Larimer and colleagues [11]. Students were asked to indicate the extent to which they felt other students would approve of i) drinking every day, ii) drinking every weekend, iii) drinking enough to pass out. For all normative questions, the reference group was ‘other students in your university, your academic year and of the same sex as you’. As the baseline survey indicated that asking students to report their own behaviour prior to perceived norms inflated normative estimates, questions on perceived norms preceded questions about students own consumption. For these, and subsequent items on alcohol consumption, a standard drink (or unit) was defined as half of a 175 ml glass of wine, half a pint of normal strength lager or a single shot of spirits, with pictorial examples representing different numbers of units presented above all questions which asked for estimates of alcohol consumption.

#### Alcohol consumption

Alcohol consumption was assessed using the Daily Drinking Questionnaire (DDQ; [34]), comprising a grid in which students are asked to indicate how many standard drinks they drank each day of a typical week. The DDQ is widely used with university students, and demonstrates similar reliability whether administered as a paper and pencil measure or in web-based format [35]. Students were asked to provide responses in relation to a typical week in the current term (January-April 2012). Units per week were calculated by summing responses for each day. An additional measure of alcohol consumption was provided by the Alcohol Use Disorders Identification Tool Consumption (AUDIT-C) scale [36], in which students indicate on 5-point likert scales how often they drink alcohol, how many units they drink on a typical occasion and how regularly they drank 6 or more units for women, or 8 or more for men. A score of 3 or more for women or 4 or more for men has been shown to optimally identify higher risk drinkers [37]. Finally, students were asked how many units they drank on their heaviest drinking occasion in the current university term.

#### Pre-university alcohol consumption

It was infeasible to obtain baseline measures, as students came into contact with the intervention on entry to university. Hence, a modified AUDIT-C asked students to indicate their drinking levels in the year prior to starting university.

#### Acceptability of objective measures of alcohol consumption (hair samples)

Students were asked whether in any future alcohol related study they would be willing to provide an anonymised hair sample for research purposes: i) without requiring payment, ii) only if paid or iii) not under any circumstances. That samples would be anonymised, and would not be analysed for substances other than alcohol, were emphasised. Students who selected Option 2 were asked to indicate how much payment they would require: i) £1-5, ii) £5-10, iii) £10-15, iv) £15-20 and v) more than £20.

### Randomisation

Blind remote randomisation was used to allocate halls to intervention or control conditions. Halls were stratified by institution and halls allocated alternately in a list ordered by size, with group allocation determined by one random number within each stratum.

### Procedures

Intervention materials, communicating messages developed from findings of a survey of first year students conducted in April 2011, were distributed in halls of residence in September 2011, and January 2012. Following ethical approval from the Cardiff University School of Social Sciences Research Ethics Committee (for the follow-up survey reported in this paper), each university was provided with copies of the questionnaire and participant information, and asked to consent to distribution. The follow-up survey was distributed in February 2012. The externally programmed web-links were emailed to nominated contacts for distribution via emails, postings on student union web pages and electronic notice boards. Regular updates were obtained from the survey company and as responses declined, distribution contacts were asked to refresh adverts and send reminder emails. Two reminders were distributed in two institutions, although one sent only one reminder due to conflict with another survey, whilst in another, delays identifying alternative distribution contacts following staff sickness meant that only one reminder was sent. Entry to a £100 prize draw (one prize in each university), was offered as an incentive. On following the web-link, students were presented with an information sheet and consent form, and could proceed only after indicating that they had read and understood the information provided, understood their right to withdraw at any time and wished to take part. The questionnaire was offered in English and Welsh. All data were collated by the market research company, who supplied an SPSS data-set. Efforts were made to boost responses through providing paper questionnaires, with questionnaires, information sheets and consent forms posted under doors in halls of residence with freepost envelopes. To discourage duplicate entries, information sheets requested that students complete either web or paper versions and emphasised that one prize draw entry per student would be accepted.

### Analysis

Response rates are broken down by institution and survey type (web / paper). In order to assess exposure and contamination, the number and percentage of students within intervention and control arms reporting seeing intervention materials in their own or another students hall are presented. Among students reporting seeing each material, percentages who accurately identify core messages are then presented. Among students who report seeing at least one material, the frequency and percentage agreeing with each statement relating to responses to and perceived impacts of the intervention are presented. Agreement scores were then correlated with pre-university drinking levels using Spearman’s rank correlation.

Due to the skewness of data relating to descriptive norms (skewness = 3.2), linear regression analyses were deemed inappropriate. Hence, descriptive norms are divided into gender-adjusted ordinal categories, including ranges used by Bewick et al. [6], and the percentage reporting a norm within each risk category presented for intervention and control participants, prior to construction of ordinal regression models [38]. Regression models adjust for age, gender, survey type (web or paper), and university. Given that this was a cluster randomised trial, a random term for hall of residence was used to account for the clustered nature of the sample. Data were also analysed using linear regression analyses of log-transformed scores. As these gave the same results as the ordinal models, only the ordinal models are reported. Injunctive norms (for which data were normally distributed) were subjected to linear regression analyses. Primary analyses were conducted on an intention-to-treat basis (i.e. comparisons were made based on the group that students were randomly allocated to, regardless of whether or not they received, or were exposed to, the intervention). Secondary ‘per-protocol’ analysis repeated the above analyses with students grouped by whether or not they had seen at least one intervention material. Intra-cluster correlations and standard deviations for total units per week are also presented. For drinking outcomes, while no formal analysis of statistical significance is conducted (as this was an exploratory trial which was not powered to definitively evaluation differences between intervention and control groups in terms of drinking behaviour) the percentages of students within intervention and control groups, and the percentage of students who had or had not seen intervention messages, reporting each level of alcohol consumption are presented. Finally percentages of students providing each response in relation to willingness to provide hair samples are presented.

## Results

### Response rates

In total, 554 students within 43 halls of residence (20 control/23 intervention) provided sufficiently complete responses (i.e. completed demographic details and a measure of perceived norms), equating to approximately 14.6% of students in the 50 halls selected for the study. Response rates were highest in sites who distributed two reminders and used email lists to target 1st years 19.5% and 25.3%, and weakest in those who distributed only 1 reminder and relied upon methods such as electronic notice boards (5.8% & 6.1%). In both intervention and control arms, 70% of responses were to the web survey, with the remainder from the paper survey. A check of email addresses provided for the prize draw revealed that no students completed both web and paper versions.

### Sample characteristics

Sample characteristics by trial arm are presented in Table 2. In both arms, more female than male responses were received. The vast majority of students were white British and home students, with a median age of 19 years. In both trial arms, the most common course types were humanities, sciences, social sciences and geography/environmental sciences. Similar scores were observed on the adapted AUDIT-C which assessed drinking levels prior to university, with means of 5.4 and 5.3 in intervention and control groups.

**Table 2:** Sample characteristics in intervention and control arms

		**Intervention**	**Control**
		**(n = 261)**	**(n = 293)**
Females		151 (57.8)	186 (63.7)
White British		232 (96.3)	257 (93.5)
Home (UK) students		236 (97.5)	263 (96.0)
Age (median)		19	19
Course type	Humanities (e.g. English)	33(12.7)	39 (13.3)
	Sciences (e.g. Physics)	31 (11.9)	40 (13.7)
	Social Sciences (e.g.	30 (11.5)	35 (12.0)
	Geography and environmental science	30 (11.5)	34 (11.6)
	Arts (Art, Music, Drama)	24 (9.2)	26 (8.9)
	Business, management	14 (5.4)	19 (6.5)
	Teacher training	8 (3.1)	11 (3.8)
	Computing and information technology	15 (5.8)	9 (3.1)
	Media studies / Journalism	3 (1.2)	8 (2.7)
	Mathematics / Statistics	9 (3.5)	7 (2.4)
	Modern languages	10 (3.9)	6 (2.1)
	Welsh	6 (2.3)	1 (0.3)
	Sport and exercise sciences	9 (3.5)	3 (1.0)
	Other healthcare subjects	4 (1.5)	2 (0.7)
	Medicine	1 (0.4)	0
	Other	33 (12.7)	53 (18.1)
AUDIT-C score – pre-university drinking (mean(SD))		5.3 (3.0)	5.4 (3.1)
Survey type completed	Web	184 (70.5)	203 (69.3)
	Paper	77 (29.5)	90 (30.7)

Figures are frequencies (and percentages) unless otherwise stated.

### Exposure and contamination

As indicated in Table 3, a large majority of students in the intervention group reported having seen posters in their own hall, with meal planners seen by a little more than half. Portable materials (i.e. those not fixed to the wall) were seen by a minority. However, there was substantial contamination, with 11-26% students in control halls seeing materials in their own hall. Large numbers of students also report having seen the materials in other students’ halls of residence. Overall, 43% of students in control halls report having seen posters, compared to 80% in the intervention group. Students were substantially more likely to report exposure to pro-alcohol promotions than to intervention materials. Overall, 429 (82.7%; 75.1% control students vs 79.8 intervention) reported having been exposed to promotions for happy hours, 430 (82.8%; 81.8% control students vs 83.6% intervention) reported exposure to promotions for drinking based events such as drink the bar dry, whilst 484 (93.4%; 92.4% control students vs 94.5% intervention) reported exposure to promotions for student drinking nights in off-campus bars and clubs.

**Table 3:** Self reported exposure to intervention materials by trial arm

**Material**	**Number (and percentage) students reporting having seen material in own hall**		**Number (and percentage) students reporting having seen material in another student’s hall**		**Number (and percentage) students reporting having seen material in either location**	
	**Intervention**	**Control**	**Intervention**	**Control**	**Intervention**	**Control**
	**(n = 240)**	**(n = 277)**	**(n = 240)**	**(n = 277)**	**(n = 240)**	**(n = 277)**
Beer mats	61 (25.5)	41 (14.8)	48 (20.5)	47 (17.1)	77 (32.7)	67 (24.2)
Mirror stickers	36 (15.1)	30 (10.8)	43 (18.4)	48 (17.5)	60 (25.5)	62 (22.4)
Posters	176 (73.6)	72 (25.9)	100 (42.6)	86 (31.3)	188 (80.0)	120 (43.3)
Meal planners	134 (56.1)	41 (14.8)	50 (21.5)	46 (16.7)	141 (60.0)	69 (25.0)
Glasses	103 (43.1)	37 (13.3)	49 (20.9)	32 (11.6)	112 (47.8)	52 (18.8)
Postcards	78 (32.6)	37 (13.3)	41 (17.5)	47 (17.1)	91 (38.7)	64 (23.2)

### Recall of messages

Among intervention students who reported seeing beer mats (n = 74), 79.7% (n = 59) correctly identified the message ‘other students drink less than you might think’, although 64.9% (n = 48) incorrectly identified the message ‘most students underestimate how much other students drink’. Of students who reported seeing the posters (n = 186), 86.6% (n = 161) correctly identified the message ‘other students drink less than you might think’, although 60.2% (n = 112) incorrectly identified the message ‘most students underestimate how much other students drink’. Of students who reported seeing the meal planners (n = 138), 19.6% (n = 24) incorrectly indicated the message that students drink alcohol most days of the week. Of students who reported seeing mirror stickers 49.2% (n = 21) correctly identified the message ‘most students disapprove of drinking everyday’. However, among students reporting seeing posters in their own hall, recall was substantially higher for intervention group students (89.0%; n = 154) than control students (50.0%; n = 35). Recall levels among students who reported seeing posters in another students’ hall, but not their own were comparable for intervention (64.7%; n = 11) and control students (68.1%; n = 35).

### Responses to and perceived impacts of intervention messages

Of intervention students who reported seeing at least one material, almost two-thirds (61.6%; n = 242) reported that messages were believable, and more than half (55.9%; n = 214) that they were relevant. Fewer (21.4%; n = 89) stated that materials influenced their perceptions of other students’ drinking. About a third (31.9%; n = 122) stated that they made them more conscious of moderating the effects of alcohol, while 13.1% (n = 50) stated that materials had impacted their own alcohol consumption. For all statements, agreement was negatively correlated with reported drinking levels prior to attending university, with perceived credibility and impacts greatest amongst students who report having been more moderate drinkers prior to intervention. Credibility (n = 383; Spearman’s rank correlation coefficient = -0.14, p < 0.01), perceived impacts on alcohol moderation behaviours (n = 383; Spearman’s rank correlation coefficient = -0.11, p = 0.02) and alcohol consumption (n = 381; Spearman’s rank correlation coefficient,= - 0.14, p < 0.01) were significantly negatively associated with past drinking, while negative associations of past drinking with perceived relevance (n = 383; Spearman’s rank correlation coefficient = -0.09, p = 0.07) and perceived impacts on perceptions of other students’ drinking (n = 381; Spearman’s rank correlation coefficient = -0.09, p = 0.08) were significant only at the 10% level.

### Impacts on normative perceptions

As indicated in Table 4, among students in intervention halls, the largest proportion reported a perceived norm for weekly consumption in the ‘hazardous’ range, whereas for students in control halls, the most common responses lay in the ‘harmful’ range. Hence, students in intervention halls appeared slightly less likely to perceive that normative drinking levels lay in the harmful range. There was little difference between groups in terms of geometric mean or median unit per week estimates. Larger differences were observed where students are classified according to exposure, with 41% of students who had not seen any of the materials reporting a perceived norm in the harmful range by comparison to 29% of students who had. Mean injunctive norm scores were 9.8 (95% CI; 9.4 to 10.3) and 10.5 (95% CI; 10.0 to 10.9) for intervention and control participants respectively. Where grouped according to reported exposure, mean scores of 10.0 (95% CI; 9.6 to 10.3) and 10.9 (95% CI; 10.2 to 11.7) were observed for those who had / had not seen the materials.

**Table 4:** Number (and percentage) of students reporting a perceived drinking norm within each gender-adjusted ordinal categories

	**Intention to treat**		**Per protocol**	
	**Intervention (n = 261)**	**Control (n = 293)**	**Seen (n = 393)**	**Not seen (n = 115)**
Within recommended limits*	48 (18.4)	57 (19.5)	78 (19.9)	13 (11.3)
Slightly hazardous**	45 (17.2)	43 (14.7)	61 (15.5)	19 (16.5)
Hazardous***	95 (36.4)	93 (31.9)	141 (35.9)	36 (31.3)
Harmful****	73 (28.0)	99 (34.0)	113 (28.8)	47 (40.9)
Geometric mean (and 95% CI)	28.0 (25.5 to 30.6)	29.1 (26.8 to 31.6)	28.0 (26.0 to 30.1)	32.9 (29.3 to 36.9)
Median units per week	30	30	30	33.5

*1-14 units for women; 1–21 for men; **15-21 for women; 22 to 28 for men; *** 22–35 for women; 29 to 50 for men; **** >35 for women; >50 for men.

Ordinal regression models for descriptive norms indicate no significant between group differences in intention-to-treat analysis (see Table 5). In per-protocol analysis, significantly lower perceived norms were reported among students reporting exposure to intervention materials, before and after adjustment for pre-university alcohol consumption. Similarly, linear regression models for injunctive norms indicate no significant between group difference in intention to treat analysis, though lower scores among students who reported having seen the materials than among those who had not.

**Table 5:** Odds ratios / b-coefficients for intervention effects on descriptive and injunctive norms

		**Intention to treat**			**Per protocol**		
		**N = 542/505**	**N = 506/504**	**p**	**N = 498**	**N = 498**	**p**
Descriptive norm – units per week*	Intervention	0.89 (0.56 to 1.42)	1.00 (0.66 to 1.51)	0.99	**0.65 (0.46 to 0.92)**	**0.61 (0.44 to 0.84)**	0.01
	Pre university alcohol consumption level		**1.22 (1.17 to 1.27)**	<0.01		**1.23 (1.18 to 1.28)**	<0.01
Injunctive norm**	Intervention	-0.80 (-1.79 to 0.20)	-0.79 (-1.76 to 0.18)	0.11	**-0.98 (-1.87 to -0.08)**	**-0.98 (-1.86 to -0.11)**	0.03
	Pre university alcohol consumption level		0.06 (-0.06 to 0.19)	0.32		0.06 (-0.06 to 0.19)	0.31

Coefficients significant at the 5% level are highlighted in bold. P-values relate to final multivariate models. All models adjust for age, sex, survey type and university. Models in 2nd and 5th column also adjusted for reported pre-university drinking level.

*Ordinal logistic regression models ** Linear regression models.

### Design parameters and impacts on consumption

A non-significant intra-cluster correlation of 0.01 was observed for log-transformed values for total units per week, indicating no significant variance at the hall level, with a standard deviation of 1.13. In both groups, approximately 7% of students reported being non-drinkers, whilst the largest proportion of students reported drinking within recommended limits. Small majorities of both groups reported drinking above recommended limits (21 weekly units for men and 14 for women). While not subjected to formal hypothesis testing, descriptive statistics indicate that there was less than 1 unit per week difference between geometric mean weekly consumption values for intervention and control groups, and between those who had or had not seen the intervention materials. Mean AUDIT-C scores, percentages of students classified as ‘higher risk’ drinkers and median units on the heaviest drinking occasion were similar for intervention and control groups, and for those who had or had not seen intervention materials (see Table 6).

**Table 6:** Number (and percentage) of students reporting a perceived drinking norm within each gender-adjusted ordinal category

	**Intention to treat**		**Per protocol**	
	**Intervention**	**Control**	**Seen**	**Not seen**
Non-drinker	16 (6.8)	19 (6.8)	28 (7.3)	6 (5.4)
Within recommended limits*	92 (39.3)	102 (36.4)	143 (37.2)	43 (38.7)
Slightly hazardous**	42 (18.0)	36 (12.9)	63 (16.4)	14 (12.6)
Hazardous***	45 (19.2)	67 (23.9)	79 (20.6)	28 (25.2)
Harmful****	39 (16.7)	56 (20.0)	71 (18.5)	20 (18.0)
Geometric mean units per week (and 95% CI) - drinkers only	17.7 (15.6 to 20.2)	18.7 (16.6 to 21.0)	18.3 (16.5 to 20.3)	17.9 (14.8 to 21.6)
Geometric mean units per week (and 95% CI)	14.8 (12.7 to 17.3)	15.6 (13.5 to 18.0)	15.0 (12.6 to 19.4)	15.7 (13.3 to 17.0)
Median units per week	18	20	18	19
Mean AUDIT C score	6.4 (6.1 to 6.8)	6.5 (6.2 to 6.9)	6.5 (6.2 to 6.8)	6.4 (5.8 to 7.1)
‘Higher risk’ drinkers	204 (86.1)	249 (87.4)	338 (86.7)	100 (87.7)
Median units on heaviest drinking occasion	17.5	16	17	17

*1-14 units for women; 1–21 for men; **15-21 for women; 22 to 28 for men; *** 22–35 for women; 29 to 50 for men; **** >35 for women; >50 for men.

**Figure 1:**
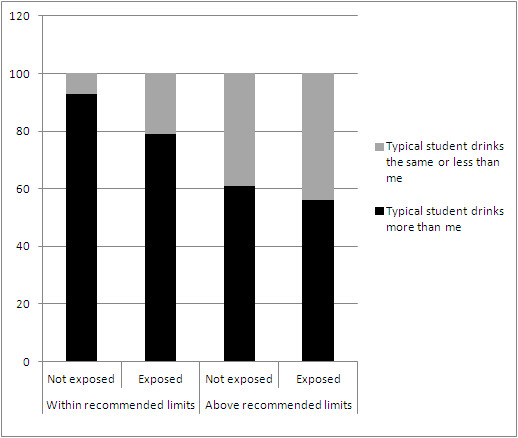
Percentage of students perceiving that a typical student drinks more than them, by alcohol consumption and intervention exposure.

Figure 1 shows the percentage of students providing estimates of ‘typical student’ consumption higher than their own (i.e. perceived that they were below average drinkers) by consumption level and exposure to intervention materials. Among students who reported drinking within recommended limits, those reporting exposure to materials were substantially less likely to perceive that a typical student drank more than them (93% vs 79%). A smaller discrepancy was observed between those exposed and not exposed to materials among students who drank in excess of recommended limits (61% vs 56%). Hence, a small majority of students drinking above recommended limits continued to perceived that they drank below typical levels.

### Acceptability of objective methods of evaluating alcohol consumption

Overall, 37.9% (n = 192) of students reported that they would provide a hair sample as an objective measure of alcohol consumption without requiring payment, while a further 46.0% (n = 233) would do so if payment were offered. Only 16.2% (n = 82) would not provide a hair sample under any circumstances. Of those students who stated that payment would be required to persuade them to provide a sample, 11.9% (n = 28) stated that £1-5 would be sufficient, 30.1% (n = 71) that they would require £5-10, 22.0% (n = 52) that they would require between £10-15, 20.8% (n = 49) that they would require £15-20, and 15.3% that they would require more than £20.

## Discussion

This study offers a range of insights into the feasibility and acceptability of implementing a social norms intervention in university halls of residence, and evaluating it using cluster randomised controlled trial methods. Key challenges which would require further attention before progression to definitive trial could be recommended, centred around difficulties in obtaining sufficient response rates to be confident of representativeness, identifying effective communication channels to achieve widespread reach of intervention messages, reducing contamination between trial arms and ensuring the credibility of key intervention messages. Ultimately, the evaluation provided tentative evidence of changes in perceived norms, though little change in behavioural outcomes. Each of these key issues is now discussed before considering implications.

Whilst exceeding the 4% response rate achieved in the only UK-based peer reviewed survey of students’ drinking norms to date [17], the response rate of 15% was perhaps insufficient to be confident of representativeness. Furthermore, rates were highly differentiated across sites; in sites achieving the best responses, bespoke email lists targeted first year students, with 2 reminder mailings sent. In sites achieving the poorest rates, distribution relied upon electronic notice boards, or email distribution via course representatives. In one, the survey was given a lower priority than an existing survey, with clashes meaning that access was denied to distribution channels such as electronic notice-boards. In another, illness of the nominated contact coincided with the start of the distribution period, with no plans made to cover this work, leading to delays in identifying alternative distribution channels. Response rates were boosted by addition of a pen and paper version of the survey, delivered by researchers where universities allowed this. In one of 2 sites who asked to arrange distribution themselves, surveys were not distributed prior to the end of term.

Most students in intervention halls reported seeing posters in their hall, though materials not affixed to the walls were seen by fewer. It is unclear whether the large proportions of students not seeing materials other than posters arose from non-placement, from not being sufficiently noticeable, or disposal by other students. Process evaluation observations reported elsewhere suggest that these factors combined. Although materials were evident throughout intervention halls at Phase 1 or 2, in some cases only one round appeared to have been placed, whilst materials such as meal planners had often been taken down and placed in recycling bins.

As argued by MacDonald and colleagues [31], for a campaign to be successful, messages must be understood, believed and viewed as relevant (although others have questioned the importance of relevance, arguing that people underestimate the influence of social norms on their behaviour [39]). Most students who reported seeing posters or drinks mats recognised descriptive norm messages, although injunctive norm messages were recognised by fewer. A slight majority perceived intervention messages as credible and relevant, though most felt that they would have little impact on behaviour. Furthermore, pre-university drinking levels and credibility were negatively correlated. This is perhaps unsurprising given that dissonance between current beliefs and normative communications is likely greater for heavier drinkers. However, it has implications for social norms interventions, in that whilst these commonly operate by targeting higher risk drinkers for feedback on ‘actual’ norms [18], such individuals appear more likely to dismiss such information as lacking credibility.

Many control students reported exposure to materials, perhaps indicating that using residence hall as the unit of randomisation may not be feasible for definitive trials of similar interventions. However, while most who reported seeing posters in another students’ hall correctly recalled their core message, only half of control participants reporting seeing the posters in their own hall did so compared to almost 90% of those in intervention halls. This perhaps indicates either misreporting of exposure among control students, consistent with researcher observations which found no intervention materials in control halls, or relatively brief exposure. Given that twice as many intervention as control participants were exposed to materials, and given the higher recall amongst ‘exposed’ members of the intervention group, while reduced by contamination, one would perhaps still expect to have seen differences in normative perceptions between intervention and control groups if effects were large.

Between group comparisons offered equivocal evidence of impacts on perceived norms. In intention-to-treat analysis, a small and non-significant reduction in estimates of typical consumption among other students was observed. Comparisons between students exposed and not exposed to materials indicated larger discrepancies, which remained significant after adjustment for demographic variables and pre-university drinking levels. However, whilst not subjected to formal hypothesis testing, between group differences in relation to post-intervention drinking levels were small, regardless of whether compared by randomisation status or exposure to materials. One explanation for limited behavioural change, even where comparing students on the basis of exposure, lay in the fact that differences in normative perceptions appeared greater for students who drank within recommended limits, consistent with findings that heavier drinkers were more likely to reject messages as untrue.

In relation to acceptability of objective measures, most reported willingness to provide hair samples as an objective measure, though half stated that they would require payment to do so. Whilst it should be noted that this percentage is based on students who have engaged with the study, and cannot be generalised beyond this subsample, high willingness to provide sample is encouraging, and suggests that it may be feasible to adopt more objective measures in order to rule out chances that between group differences arise from differential reporting biases.

Limitations of this study include that only 4 universities were recruited, and whether feasibility challenges encountered would be reflected to a greater or lesser degree in a national roll-out is unclear. It may have been that universities which agreed to take part were more receptive to alcohol interventions than those who were not, or that drinking levels in these universities differed from those in other sites. Furthermore, consistent with previous UK university based alcohol surveys [6,17], the study recruited more female than male undergraduates. Achieving higher overall response rates, and responses more representative in terms of factors such as gender, may be necessary to understand the reach and effects likely to be achieved by such campaigns. The development of the campaign was hampered by the fact that policy timetables did not allow for qualitative research with students to develop relevant and credible messages prior to implementation. Qualitative data conducted as part of the process evaluation, to be reported elsewhere, highlight a number of issues in relation to, which ideally would have been explored in developmental phases. Furthermore, outcomes are based on self-report. Nevertheless, the study benefitted from the fact that it was a pragmatic evaluation of a real world intervention, therefore offering high external validity, and highlighted challenges in relation to implementation and receipt often overlooked in social norms evaluations. Its findings demonstrate the importance of encouraging policy and practice partners to use exploratory trials prior to embarking on full-scale roll-outs or expensive definitive trials, and highlight a number of uncertainties and challenges which would need to be overcome in any definitive evaluation of a similar intervention.

## Conclusions

In terms of intervention delivery and content, significant development appears to be needed before definitive evaluation can be considered. There is perhaps a need for more prolonged efforts to engage with universities, maximise buy-in and improve local ownership of the intervention. Greater efforts are needed to enhance the visibility, credibility and relevance of social norms intervention messages, particularly amongst more hazardous drinkers who were more likely to dismiss messages as untrue. Whilst intention-to-treat analysis indicated no effect on perceived norms, there is tentative suggestion of reductions in perceived drinking norms among students exposed to the social norms intervention. This non-randomised secondary analysis should be interpreted with the caveat that those exposed to the intervention may have differed from those not exposed in other ways which influence these perceptions. Furthermore, impacts on behaviour appear negligible, with little difference between groups even where participants were grouped by exposure.

In relation to evaluation design, establishing effectiveness would require significant improvements in response rates. The study was clearly in some cases given lower priority than other activities, such as annual student surveys, and a longer time period may be needed to build relationships with universities, enhance buy-in, and negotiate more effective distribution methods. Given the greater effectiveness of bespoke email lists in eliciting student responses, by comparison to electronic notice-boards, a definitive trial should aim to negotiate construction of email lists to target first year students in all sites, perhaps requiring funding to incentivise their creation. Another means of boosting response rates is use of incentives. Whilst entry to a £100 prize draw was offered, many US based social norms surveys achieving response rates closer to 40% have included payment in the region of $10 for each participant [15,19]. Offering £5 per student would have inflated the cost of this study by approximately £8000 if this were to achieve a 40% response rate, with this figure increasing in line with the increase in size of a fully powered trial. Using cluster randomisation at the hall-level appears associated with contamination between trial arms, although given that recall of key messages was so much poorer among control students than intervention group students, one would perhaps still expect to see between group differences if intervention effects were large. Research is needed to evaluate the acceptability and response rates achieved in practice where attempting to obtain hair samples from students as an objective measure of consumption.

## Competing interests

The authors declare that they have no competing interests.

## Authors’ contributions

GM led the development, distribution and analysis of survey measures and drafted the paper. All authors contributed to trial design, commented on drafts and agreed the final manuscript.
